# Comparative metagenomic and metatranscriptomic analyses reveal the role of the gayal rumen and hindgut microbiome in high-efficiency lignocellulose degradation

**DOI:** 10.1186/s40104-025-01335-1

**Published:** 2026-02-02

**Authors:** Shijia Li, Jiawei Zhang, Lin Han, Ye Yu, Abdallah A. Mousa, Weiyun Zhu, Jing Leng, Fei Xie, Shengyong Mao

**Affiliations:** 1 Centre for Ruminant Nutrition and Cleaner Production Innovation, College of Animal Science and Technology, Nanjing Agricultural University, Nanjing, China; 2 Laboratory of Gastrointestinal Microbiology, Jiangsu Key Laboratory of Gastrointestinal Nutrition and Animal Health, National Center for International Research On Animal Gut Nutrition, College of Animal Science and Technology, Nanjing Agricultural University, Nanjing, China; 3 Faculty of Animal Science and Technology, Yunnan Agricultural University, Kunming, China; 4 Key Laboratory of Animal Nutrition and Feed Science of Yunnan Province, Yunnan Agricultural University, Kunming, China

**Keywords:** Auxiliary activity enzymes, Gayal, Lignocellulose, Rumen and hindgut microbiome, Yellow cattle

## Abstract

**Background:**

The gayal (*Bos frontalis*), a semi-domesticated bovine species, demonstrates exceptional adaptability to lignocellulose-rich diets dominated by bamboo, suggesting the presence of a specialized gastrointestinal microbiome. However, the functional mechanisms underlying this host-microbiome interaction remain poorly understood. Here, we conducted integrated metagenomic and metatranscriptomic analyses of rumen, cecum, and colon digesta from yellow cattle and gayal raised on the same bamboo-based high-fiber diet.

**Results:**

The results showed that gayal exhibited superior fiber-degrading capacity relative to yellow cattle, evidenced by significantly higher (*P* < 0.05) fiber digestibility, cellulase and xylanase activities, and increased volatile fatty acids production despite identical feed intake. Microbial community analysis revealed distinct composition in both the rumen and hindgut of gayal compared to yellow cattle, with notable enrichment of taxa specialized in lignocellulose degradation. Metatranscriptomic profiling further identified upregulation of key lignin-modification enzymes, particularly AA6, AA2, and AA3, primarily encoded by *Prevotella*, *Cryptobacteroides*, *Limimorpha*, and *Ventricola*. These enzymes are known to modify lignin structure to increase polysaccharide accessibility. These results demonstrate that gayal hosts a unique and metabolically active gastrointestinal microbiome capable of efficient lignocellulose deconstruction through a coordinated enzymatic cascade, especially effective in dismantling lignin barriers.

**Conclusions:**

This study provides novel insights into host-microbiome co-adaptation to fibrous feeds and highlights the potential of gayal-derived microbial consortia and enzymes for improving roughage utilization in ruminant agriculture.

**Supplementary Information:**

The online version contains supplementary material available at 10.1186/s40104-025-01335-1.

## Introduction

Lignocellulose, the primary structural component of plant cell walls, is the most abundant renewable biomass on Earth. However, its complex and highly cross-linked structure poses significant challenges for efficient degradation. Herbivores have evolved symbiotic microbial communities in their digestive tracts to overcome this recalcitrance, making these systems a critical focus of research on lignocellulose degradation mechanisms [1–4]. Over the past decades, microbial communities of gastrointestinal fermenters, particularly domesticated ruminants such as dairy cattle, water buffalo, sheep, and goats [5–8], have been extensively studied. Studies on these species have revealed a complex division of work within the rumen consortium. Bacterial strategies for lignocellulose deconstruction include the cellulosome complexes produced by *Ruminococcus flavefaciens* [9], the highly efficient cellulolytic machinery of *Fibrobacter succinogenes* [10], and the polysaccharide utilization loci (PULs) predominantly from Bacteroidetes [11]. Additionally, anaerobic fungi such as *Neocallimastix* and *Piromyces* not only secrete free enzymes and form cellulosomes distinct from bacteria [12, 13], but also physically penetrate the lignocellulosic matrix through their hyphal rhizoid system [14]. Further ecosystem complexity is contributed by protozoa, which modulate bacterial community dynamics through predation, and by archaea, which enhance fermentation efficiency by consuming hydrogen to maintain a low partial pressure [15, 16]. However, one rare and unsystematically domesticated ruminant species, the gayal (*Bos frontalis*), which exhibits exceptional lignocellulose utilization efficiency, remains understudied.

Native to the Dulong River and Nujiang River basins in Yunnan Province, China, the gayal thrives on a bamboo-dominated diet, a trait not commonly observed in other cattle species. Bamboo is a particularly challenging food source, composed of 70%–80% lignocellulose with low nutritional and protein content [17, 18], posing a significant physiological hurdle for most mammals. Although some studies have explored the gut microbiota of specialized bamboo feeders such as giant pandas and bamboo rats, the microbial mechanisms underlying efficient bamboo digestion in gayal remain poorly understood [17–20]. Interestingly, genomic, behavioral, and growth performance studies have revealed significant distinctions between gayal and conventional cattle breeds [21–23]. These unique features suggest that the gayal’s gastrointestinal microbiota may harbor specialized adaptations tailored to bamboo digestion, highlighting the need for further investigation into these distinctive species and their natural ecological adaptations.

To elucidate the enzymatic strategies employed by the gayal’s gastrointestinal microbiota in degrading plant cell walls, we conducted a systematic multi-omics investigation. Using an integrated metagenomic and metatranscriptomic approach, we compared the composition, structure, and function profiles of rumen, cecum, and colon microbiota between yellow cattle and gayal that were fed an identical high-lignocellulose diet, thereby revealing gayal-specific gastrointestinal microbiome’s adaptation to this lignocellulose-rich regimen. Our findings further identify that the gayal rumen and hindgut microbiota constituted an underexplored reservoir of carbohydrate-processing enzyme systems, expanding our understanding of microbial strategies for deconstructing recalcitrant plant biomass. Our work offers pivotal implications for the high-value utilization of lignocellulose in agricultural and bioprocessing industries, and provides a scientific basis for the conservation and sustainable utilization of this rare species.

## Materials and methods

### Animals and experimental design

The experiment was conducted in the Phoenix Mountain Gayal Breeding and Expansion Base, situated at an elevation of 2,700 m, in Lushui City, Nujiang Prefecture, Yunnan Province, China. Sixteen healthy, two-year-old male animals were enrolled and matched for age and body weight, including eight wild gayal (*Bos frontalis*, 241.3 ± 8.97 kg) and eight local Yunnan yellow cattle (*Bos taurus*, 232.1 ± 18.3 kg). All animals were individually housed in pens and received the same high-lignocellulose diet (80% bamboo; the diet composition is shown in Table S1) throughout the entire trial. The study comprised a 7-day adaptation period followed by a 21-day experimental period. Cattle were fed three times daily (07:00, 13:00, and 19:00), and individual feed intake was recorded at each feeding. All cattle were allowed to graze freely and had ad libitum access to water and salt blocks. At the end of the trial, all animals were fasted overnight and then humanely euthanized.

### Measurement of growth performance and apparent digestibility

On both the first and final days of the experimental period, all animals were weighed before the morning feeding to determine average daily gain (ADG). During the last three days of the experimental period, daily samples of diet and residues were collected. Fecal samples were collected twice daily (morning and evening), weighed, thoroughly homogenized, and immediately frozen at −20 °C. All feed and fecal samples were oven-dried at 65 °C for 48 h and ground through a 1-mm sieve for subsequent chemical analysis. Dry matter (DM), crude protein (CP), ether extract (EE), crude ash, neutral detergent fiber (NDF), acid detergent fiber (ADF), and acid detergent lignin (ADL) were analyzed. Organic matter (OM) was calculated as DM minus crude ash. Analyses of DM, CP, EE, and crude ash in feed and fecal samples were conducted following the methods of AOAC (2012). Determination of NDF, ADF, and ADL was performed with the addition of sodium sulfite and heat-stable α-amylase [24]. Apparent digestibility was calculated using acid-insoluble ash as an internal marker, based on nutrient intake and fecal output. 

### Measurement of fermentation parameters in the rumen and hindgut

The rumen, cecum, and colon were separated immediately and their contents were collected. The pH of the contents was measured using a calibrated portable pH meter HI 9024 C (Hanna Instruments Inc., Woonsocket, RI, USA). A portion of the rumen content was centrifuged at 13,800 × *g* for 10 min at 4 °C. The supernatant was collected and stored at −20 °C for subsequent analysis of volatile fatty acid (VFA) concentrations using a GC-14B gas chromatography (Shimadzu Corporation, Kyoto, Japan) [25]. Additionally, 1 g of cecum/colon digesta was homogenized with 2 mL of deionized water and centrifuged at 2,000 × *g* for 15 min at 4 °C. The resulting supernatant was also stored at −20 °C for VFA analysis. The capillary column of gas chromatography was 30 m × 0.32 mm × 0.25 mm, with the column temperature at 110 °C, the vaporization chamber temperature at 180 °C, and the detection chamber temperature at 180 °C. The remaining contents were thoroughly mixed and frozen in liquid nitrogen for microbial DNA and RNA extraction.

### Measurement of enzyme activity in the rumen and hindgut

Samples of rumen fluid, cecum, and colon contents were processed to obtain total soluble enzymes as follows. The filtered samples were homogenized in an extraction buffer (1:10, w/v), and then sonicated on ice to release both extracellular and intracellular enzymes. The mixtures were centrifuged at 15,000 × *g* for 10 min at 4 °C to pellet insoluble material. The clear supernatant was collected and kept on ice for immediate assay. Enzyme activities of cellulase (carboxymethyl cellulase) and xylanase were determined using a commercial assay kit (Beijing Boxbio Science & Technology Co., Ltd., Beijing, China). The activities of mannosidase and arabinofuranosidase were determined using a commercial assay kit (Shanghai Enzyme-linked Biotechnology Co., Ltd., Shanghai, China). All assays were carried out according to the manufacturer’s instructions. Briefly, the supernatant was incubated with the specific substrate at their respective recommended temperature: 50 °C for xylanase, 40 °C for cellulase, and 37 °C for mannosidase and arabinofuranosidase. The final product was then quantified by spectrophotometry at the following wavelengths: 540 nm for cellulase and xylanase, 405 nm for mannosidase, and 400 nm for arabinofuranosidase.

### Microbial DNA and RNA extraction

Content samples were thoroughly homogenized using a vortex mixer. Then, 200 mg of the homogenate was combined with 100 mg of zirconia beads (0.1 mm) and 1 mL of Inhibit EX buffer in a centrifuge tube. Samples were disrupted with a bead beater (Mini-Beadbeater1, USA) at 4,800 r/min for 20 s per cycle, repeated four cycles in total. Microbial genomic DNA was extracted and purified using the QIAamp Fast DNA Stool Mini Kit (Qiagen, Germany) following Yu and Morrison [26]. DNA quality was assessed by a Qubit 4.0 fluorometer (Invitrogen, Carlsbad, CA, USA) and 1% agarose gel electrophoresis. A_260/280_ ratios were maintained between 1.95 and 2.10, measured on a ND-2000 spectrophotometer (NanoDrop Technologies, Wilmington, DE, USA). DNA samples that passed quality control were stored at −80 °C. Total RNA was extracted from digesta using TRIzol^®^ Reagent according to the manufacturer’s instructions (Invitrogen, Carlsbad, CA, USA), and genomic DNA was removed using DNase I (TaKara Bio Inc., Kusatsu, Japan). Then, RNA quality was determined using the 2100 Bioanalyzer (Agilent Technologies, Santa Clara, CA, USA) and quantified using the ND-2000 (NanoDrop Technologies, Wilmington, DE, USA). High-quality RNA samples (OD_260_/OD_280_ = 1.8–2.2, OD_260_/OD_230_ ≥ 2.0, RIN ≥ 6.5, 28S/18S ≥ 1.0, and total amount > 10 μg) were used to construct sequencing library.

### Metagenomic and metatranscriptomic sequencing and analysis

Genomic DNA was randomly fragmented into 300 bp using a Covaris M220 (Covaris, Woburn, MA, USA). Shotgun metagenomic sequencing libraries were prepared using the TruSeq DNA PCR-Free Library Preparation Kit (Illumina, San Diego, CA, USA) with a 350 bp insert and were sequenced on an Illumina NovaSeq 6000 platform. The sequencing was performed in paired-end 150 bp mode, generating over 50 Gb of data per sample. Metatranscriptomic libraries were constructed using the TruSeq Stranded Total RNA Library Preparation Kit (Illumina, San Diego, CA, USA) following the manufacturer’s protocol (cDNA synthesis, end repair, adapter ligation, and library enrichment), assessed on an Agilent 2100 Bioanalyzer (Agilent Technologies, Santa Clara, CA, USA), and sequenced on the Illumina NovaSeq 6000 platform (paired-end 150 bp; > 50 Gb per sample).

Adapter and low-quality sequences of raw reads from both metagenomic and metatranscriptomic sequencing were trimmed using the Trimmomatic [27] (v.0.33). Host and dietary reads were removed by mapping to the genomes of gayal (*Bos frontalis*, GCA_043643345.1), yellow cattle (*Bos taurus*, GCF_002263795.3), corn (*Zea mays*, GCA_902167145.1), bamboo (*Dendrocalamus latiflorus*, GCA_017311315.1), and human (*Homo sapiens*, GCA_000001405.28) using the BWA-MEM [28] (v.0.7.17). Additionally, rRNA contamination in metatranscriptomic reads was filtered using the SortMeRNA [29] (v.4.3.6). High-quality reads from each metagenomic and metatranscriptomic sample were assembled individually using the MEGAHIT [30] (v.1.1.1, parameters “--min-contig-len 500”). The statistical details of the raw data, high-quality reads, and assembly results are provided in Table S5. Open reading frames (ORFs) were predicted on contigs using the Prodigal [31] (v.2.6.3, parameters “-p meta”). ORFs > 100 bp were retained and clustered based on nucleotide identity using the CD-HIT [32] (v.4.8.1, parameters “-n 9 -g 1 -c 0.95 -G 0 -M 0 -d 0 -aS 0.9”). After removing redundant genes (≥ 95% nucleotide sequence identity and ≥ 90% coverage) [33], a nonredundant gene catalog was constructed. Separate nonredundant gene catalogs were constructed for the rumen, cecum, and colon microbiomes, comprising 47,174,431, 33,515,982, and 30,067,327 genes, respectively.

The nonredundant gene catalog was annotated by aligning predicted proteins against the NCBI-NR using the DIAMOND BLASTP [34] (v.0.9.22). For each protein, the top-scoring homologous hit was retained for taxonomic and functional assignment. Sequences with tied scores were manually verified to exclude potential misassignments. For KEGG annotation, we employed KofamScan [35] (v.1.3.0) to assign KEGG Orthology (KO) identifiers to the protein sequences, and the resulting KO identifiers for each sample were then mapped against the KEGG PATHWAY database (https://www.genome.jp/kegg/pathway.html) [36] to reconstruct metabolic pathways. Carbohydrate-active enzymes (CAZymes) were identified by querying protein sequences against the CAZy database [37] with the HMMER [38] (v.3.2.1) using profile hidden Markov models. To quantify gene abundance, high-quality reads from each sample were mapped to the gene catalog using the BWA-MEM [28] (v.0.7.17) (alignment length ≥ 50 bp and identity > 95%). Gene abundances were computed as transcripts per million (TPM), and the abundances of taxa, KOs, and CAZymes were obtained by summing TPM values across genes assigned to each category [33].

### Statistical analysis

Growth performance, apparent digestibility, fermentation parameters, and enzymatic activity were compared between yellow cattle and gayal using the Independent Samples *t*-test. For taxonomic and functional levels, the Bray–Curtis distance matrices [39] of the rumen microbiota from yellow cattle and gayal were subjected to ordination analysis. Group differences were assessed with ANOSIM (9,999 permutations) using the R package vegan [40] (v.2.6–4), and results were visualized with principal coordinates analysis (PCoA). Alpha diversity (Shannon index) for the microbial communities between the two breeds was calculated using the R vegan package [40] (v.2.6–4). The Wilcoxon rank-sum test was used to compare taxonomic and functional profiles between yellow cattle and gayal. Statistical significance was defined as *P* < 0.05 for all tests.

## Results

### Growth performance, apparent digestibility, and fermentation parameters of yellow cattle and gayal

**Fig. 1:**
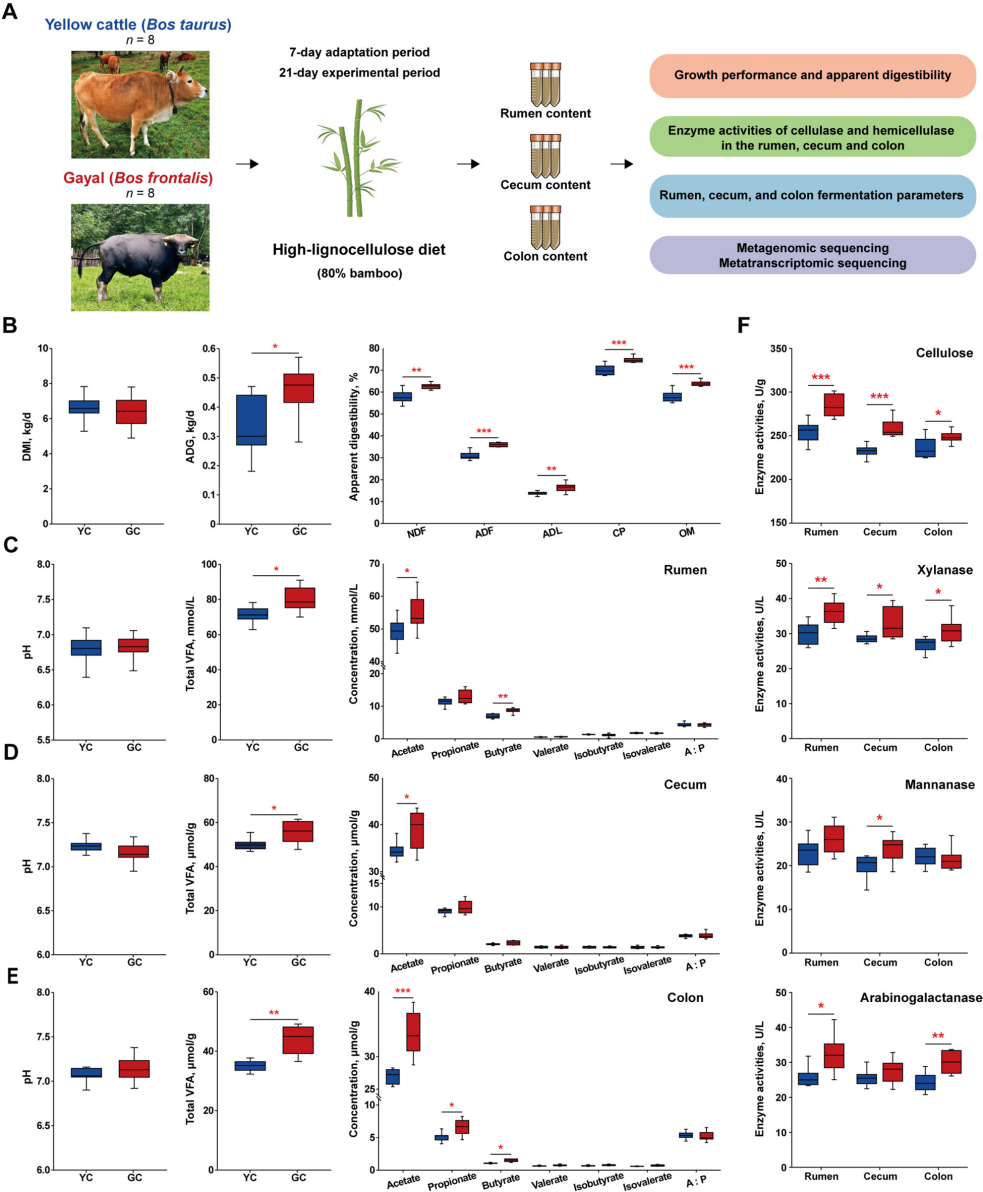
Comparison of growth performance, apparent digestibility, and fermentation parameters of rumen, cecum and colon between yellow cattle (YC) and gayal (GC). **A** Schematic overview of the experimental design. **B** Growth performance and apparent digestibility of YC and GC. **C–E ** Fermentation parameters in the rumen (**C**), cecum (**D**), and colon (**E**), including pH and VFA concentrations. **F** Cellulase, xylanase, mannanase, and arabinogalactanase activities in the rumen, cecum, and colon. Data are presented using box plots, where the center line represents the median, the box indicates the interquartile range (IQR), and whiskers extend to 1.5 × IQR. Significance levels between groups are indicated as follows: ^*^P < 0.05, ^**^P < 0.01, and ^***^P < 0.001

Under identical dietary conditions (Fig. 1A), gayal showed no significant difference in dry matter intake (DMI) compared with yellow cattle, but exhibited a significantly greater ADG (*P* = 0.034; Fig. 1B; Table S2). Furthermore, gayal demonstrated higher digestibility of NDF (*P* = 0.002), ADF (*P* < 0.001), ADL (*P* = 0.005), CP (*P* < 0.001), and (OM (*P* < 0.001) (Fig. 1B; Table S2). Rumen fermentation analysis revealed that gayal had significantly higher concentrations of total VFAs (*P* = 0.012), acetate (*P* = 0.043), and butyrate (*P* = 0.001) (Fig. 1C; Table S3). A similar trend was observed in the cecum, where concentrations of total VFAs (*P* = 0.028), and acetate (*P* = 0.034) were also significantly elevated in gayal compared to yellow cattle (Fig. 1D; Table S3). Furthermore, in the colon, gayal showed significantly higher concentrations of total VFAs (*P* = 0.001) and acetate (*P* < 0.001) compared to yellow cattle, consistent with the patterns observed in the rumen and cecum. Additionally, significant differences were also found in propionate (*P* = 0.016) and butyrate (*P* = 0.01) concentrations in the colon (Fig. 1E; Table S3). The elevated VFA concentrations across gastrointestinal regions suggested enhanced microbial fermentation activity, which may be driven by differences in key carbohydrate-degrading enzymes. Specifically, rumen cellulase (*P* < 0.001), xylanase (*P* = 0.005) and arabinogalactanase (*P* = 0.015) activities were significantly greater in gayal than in yellow cattle (Fig. 1F; Table S4). Likewise, in the cecum and colon, cellulase (*P* < 0.001) and xylanase (*P* = 0.018) activities were all higher in gayal, whereas mannanase activity was higher in the gayal cecum (*P* = 0.024), and arabinogalactanase activity was higher in the colon (*P* = 0.002) (Fig. 1F; Table S4). These results collectively indicated that gayal possesses a superior capacity to degrade and utilize lignocellulose compared with yellow cattle, which likely contributes to their improved growth performance and gastrointestinal fermentation efficiency.

### Community structure and diversity in the rumen and hindgut microbiota of yellow cattle and gayal

**Fig. 2:**
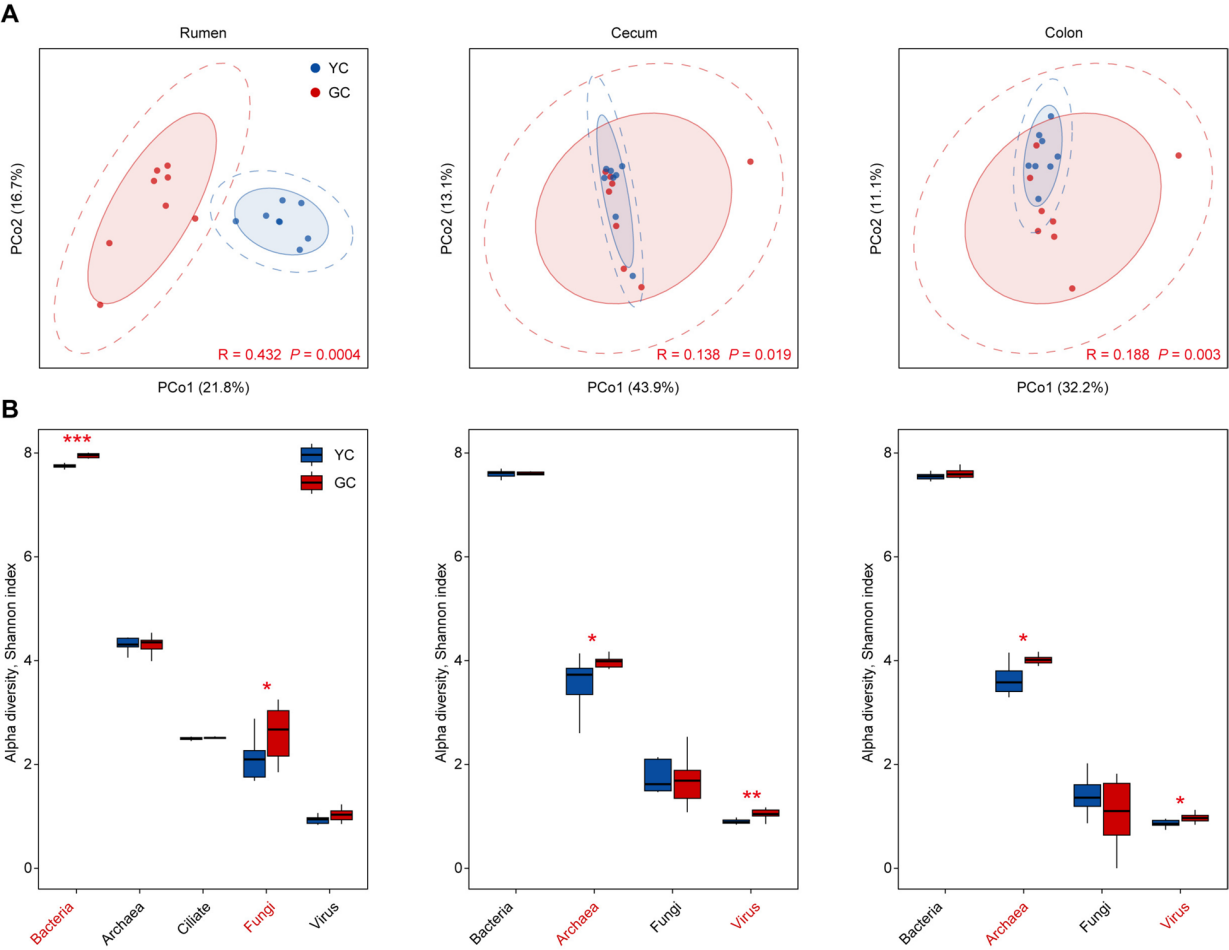
Structural and functional divergence of microbiota in the rumen, cecum and colon between yellow cattle (YC) and gayal (GC). **A** Principal coordinates analysis (PCoA) based on species-level metagenomic profiles of the rumen, cecum, and colon microbiota. **B** Comparison of microbial alpha diversity between YC and GC. Data are presented using box plots, where the center line represents the median, the box indicates the interquartile range (IQR), and whiskers extend to 1.5 × IQR. Significance levels between groups are indicated as follows: ^*^P < 0.05, ^**^P < 0.01, and ^***^P < 0.001

Principal coordinates analysis (PCoA) of species-level metagenomic profiles revealed significant structural divergence in the rumen (R = 0.432, *P* < 0.001), cecum (R = 0.138,* P* = 0.019), and colon (R = 0.188,* P* = 0.003) microbiota between yellow cattle and gayal, with distinct clustering patterns corresponding to host species (Fig. 2A). Further analysis of the rumen microbiota showed significant breed-associated differences in bacterial (*P* < 0.001) and fungal (*P* = 0.034) communities, with bacteria (R = 0.432) exhibiting greater dissimilarity than fungi (R = 0.214). In contrast, archaeal, ciliate, and viral communities did not differ significantly between yellow cattle and gayal (Fig. S1). Within the cecum, significant differences were observed in bacteria (*P* = 0.021) and archaea (*P* = 0.016), with archaeal communities exhibiting greater dissimilarity (R = 0.229) than bacterial communities (R = 0.137), whereas fungal and viral communities showed no significant variation (Fig. S2A). Similarly, analysis of the colon microbiota revealed significant breed-specific differences in bacterial (*P* = 0.004), archaeal (*P* = 0.007), and fungal (*P* = 0.016) communities, with archaea exhibiting greater dissimilarity (R = 0.342) than bacteria (R = 0.181) and fungi (R = 0.179), while viral communities did not differ significantly between breeds (Fig. S2B). Alpha diversity analysis further revealed that gayal harbored significantly higher microbial diversity for bacterial (*P* = 0.001) and fungal (*P* = 0.05) communities in the rumen and for archaeal (*P* = 0.021 and *P* = 0.021) and viral (*P* = 0.007 and *P* = 0.028) communities in the cecum and colon (Fig. 2B). Collectively, these findings suggested that the enhanced lignocellulose degradation capability in gayal is associated with its distinct gastrointestinal microbiota, with fungi and prokaryotes playing key roles in the rumen and hindgut [41].

### Taxonomy composition of microbiota in the rumen and hindgut of yellow cattle and gayal

**Fig. 3:**
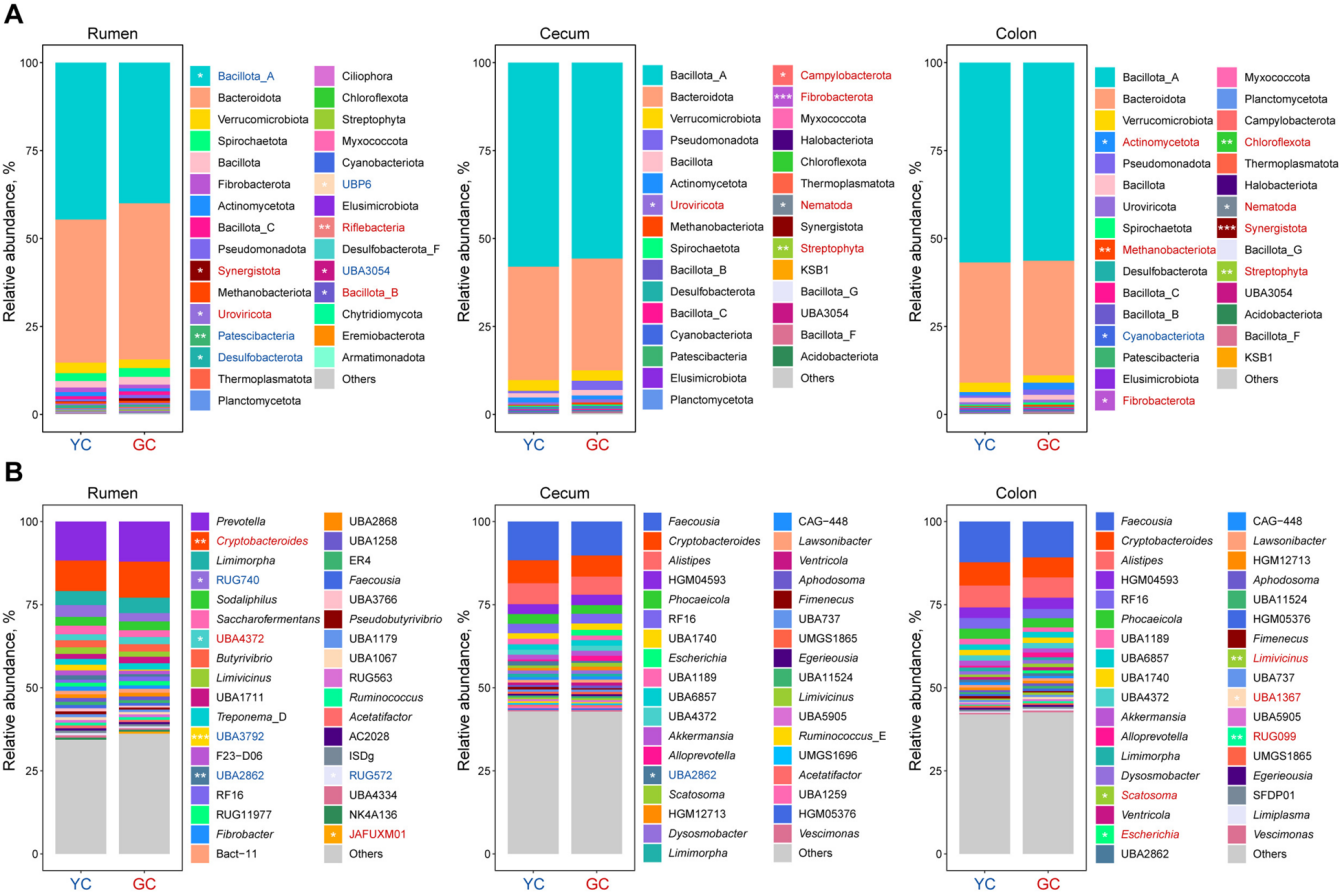
Differential taxonomic composition of microbiota in the rumen, cecum and colon between yellow cattle (YC) and gayal (GC). **A** Relative abundance of top 30 abundant microbial phyla across the rumen, cecum, and colon. **B** Relative abundance of top 35 abundant microbial genera. Taxa shown in red indicate higher relative abundance in GC, while those in blue indicate higher relative abundance in YC. Statistical significance is determined using the Wilcoxon rank-sum test (^*^P < 0.05, ^**^P < 0.01, and ^***^P < 0.001). Only significantly different taxa are marked

Comparative analysis of rumen microbiota at the phylum level revealed significant differences in the dominant taxa between yellow cattle and gayal, with gayal showing significantly higher relative abundances of Synergistota (*P* = 0.01), Uroviricota (*P* = 0.05), Riflebacteria (*P* = 0.002), and Bacillota_B (*P* = 0.038) phyla, whereas yellow cattle exhibited greater abundance of Bacillota_A (*P* = 0.05), Patescibacteria (*P* = 0.003), Desulfobacterota (*P* = 0.028), UBP6 (*P* = 0.01), and UBA3054 (*P* = 0.015) phyla (Fig. 3A; Table S6). Genus-level analysis found that gayal had significantly higher relative abundances of *Cryptobacteroides* (*P* = 0.007), UBA4372 (*P* = 0.05), and JAFUXM01 (affiliated with Synergistales; *P* = 0.01), while yellow cattle were enriched in RUG740 (*P* = 0.028), UBA3792 (*P* < 0.001), UBA2862 (*P* = 0.001), and RUG572 (*P* = 0.028) (Fig. 3B; Table S9). In the cecum, the phyla Uroviricota (*P* = 0.028), Campylobacterota (*P* = 0.028), Fibrobacterota (*P* < 0.001), Nematoda (*P* = 0.038), and Streptophyta (*P* = 0.005) were significantly more abundant in gayal (Fig. 3A; Table S7), whereas yellow cattle exhibited a higher relative abundance of the UBA2862 (*P* = 0.015) genus (Fig. 3B; Table S10). Within the colon, gayal displayed higher abundances of Actinomycetota (*P* = 0.028), Methanobacteriota (*P* = 0.007), Fibrobacterota (*P* = 0.028), Chloroflexota (*P* = 0.005), Nematoda (*P* = 0.01), Synergistota (*P* < 0.001), and Streptophyta (*P* = 0.005) phyla, while the Cyanobacteriota (*P* = 0.05) phylum was more abundant in yellow cattle (Fig. 3A; Table S8). Genus-level analysis in the colon further revealed that *Scatosoma* (*P* = 0.038), *Escherichia* (*P* = 0.05), *Limivicinus* (*P* = 0.002), UBA1367 (*P* = 0.028), and RUG099 (*P* = 0.005) were more prevalent in gayal (Fig. 3B; Table S11). In addition, we characterized the microbiota through a finer-scale taxonomic profile, categorizing it into Bacteria, Archaea, Fungi, and Protozoa (Fig. S3). While fungi and protozoa were detected, their relative abundance was substantially lower than that of bacteria.

### Metabolic functions of the rumen and hindgut microbiomes in yellow cattle and gayal

**Fig. 4:**
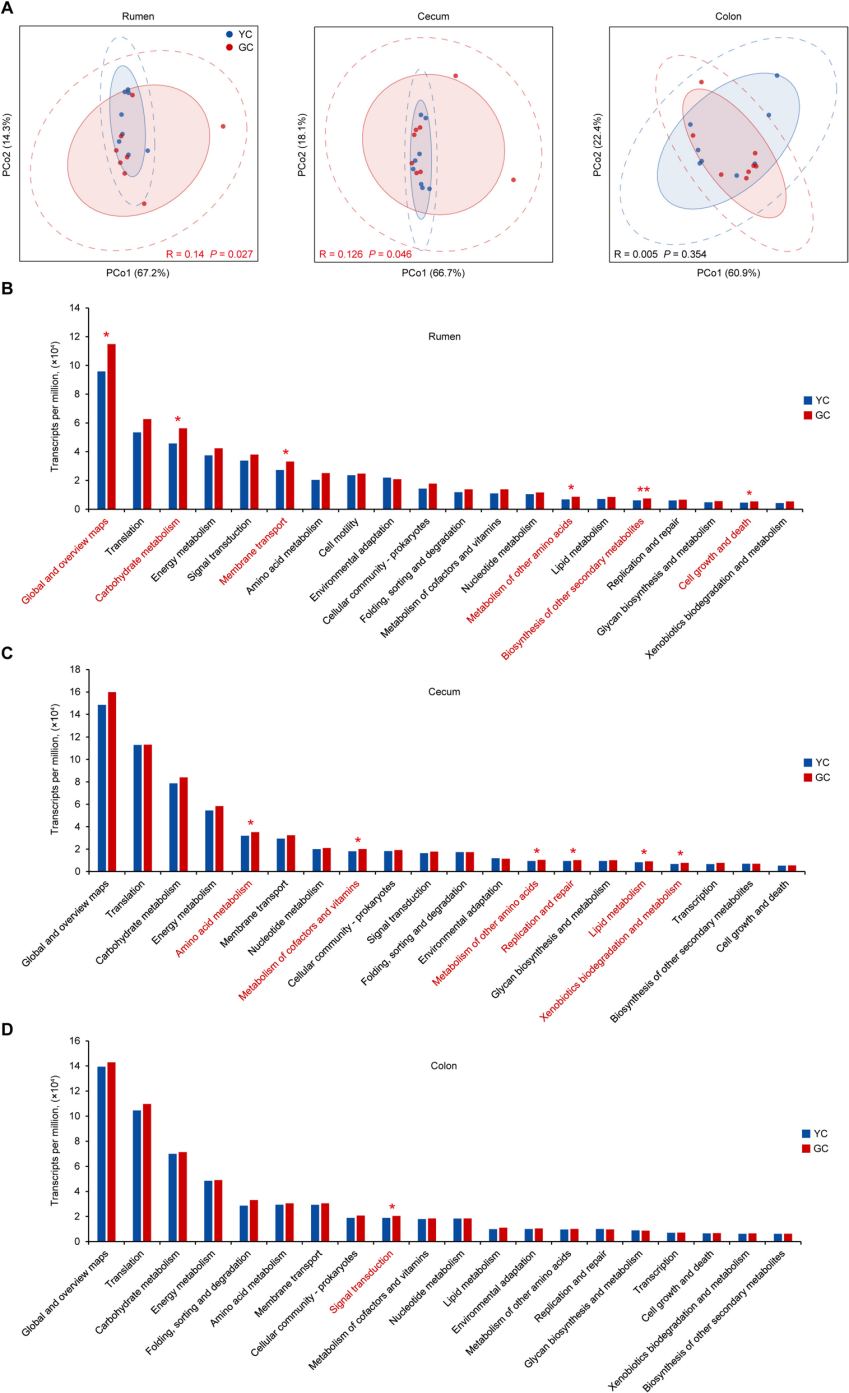
Functional divergence of metabolic pathways of microbiota in the rumen, cecum and colon between yellow cattle (YC) and gayal (GC). **A** PCoA of metatranscriptomic profiles for functional divergence based on the KO genes between YC and GC. **B**–**D **Enrichment analysis of KEGG pathways that were significantly differentially abundant in the rumen (**B**), cecum (**C**), and colon (**D**). Statistical significance is determined using the Wilcoxon rank-sum test (^*^P < 0.05, ^**^P < 0.01)

To investigate whether the observed taxonomic differences translated into functional divergence, we profiled the metabolic potential and activity of the microbiomes. Clustering of all microbial genes from the rumen, cecum, and colon revealed that the rumen contained 47,174,431 non-redundant genes, the cecum contained 33,515,982, and the colon contained 30,067,327. Among these, 18,066,893 genes in the rumen, 14,492,939 in the cecum, and 13,212,665 in the colon were annotated as involved in metabolic processes based on the KEGG pathway database. Metatranscriptomic profiling further revealed significant functional divergence between yellow cattle and gayal in both the rumen (R = 0.14, *P* = 0.027) and cecum (R = 0.126, *P* = 0.046) microbiomes (Fig. 4A). Differential KEGG pathway analysis further indicated that the rumen microbial communities of gayal were primarily enriched in global and overview maps (1.2-fold higher; *P* = 0.05), carbohydrate metabolism (1.2-fold higher; *P* = 0.021), membrane transport (1.2-fold higher; *P* = 0.05), metabolism of other amino acids (1.3-fold higher; *P* = 0.021), biosynthesis of other secondary metabolites (1.2-fold higher; *P* = 0.003), as well as cell growth and death (1.2-fold higher; *P* = 0.038) (Fig. 4B; Table S12). In the cecum, microbial communities were mainly enriched in amino acid metabolism (1.1-fold higher; *P* = 0.05), metabolism of cofactors and vitamins (1.1-fold higher; *P* = 0.015), metabolism of other amino acids (1.1-fold higher; *P* = 0.038), replication and repair (1.1-fold higher; *P* = 0.05), lipid metabolism (1.1-fold higher; *P* = 0.015), and xenobiotics biodegradation and metabolism (1.1-fold higher; *P* = 0.01) (Fig. 4C; Table S13). Conversely, the colon microbial communities of gayal showed primary enrichment in signal transduction (1.1-fold higher; *P* = 0.021) (Fig. 4D; Table S14). These metabolic specializations collectively indicate that the rumen microbiome of gayal has evolved adaptive metabolic processes tailored to its distinct dietary niche.

### Differential expression of carbohydrate-active enzymes in yellow cattle and gayal

**Fig. 5:**
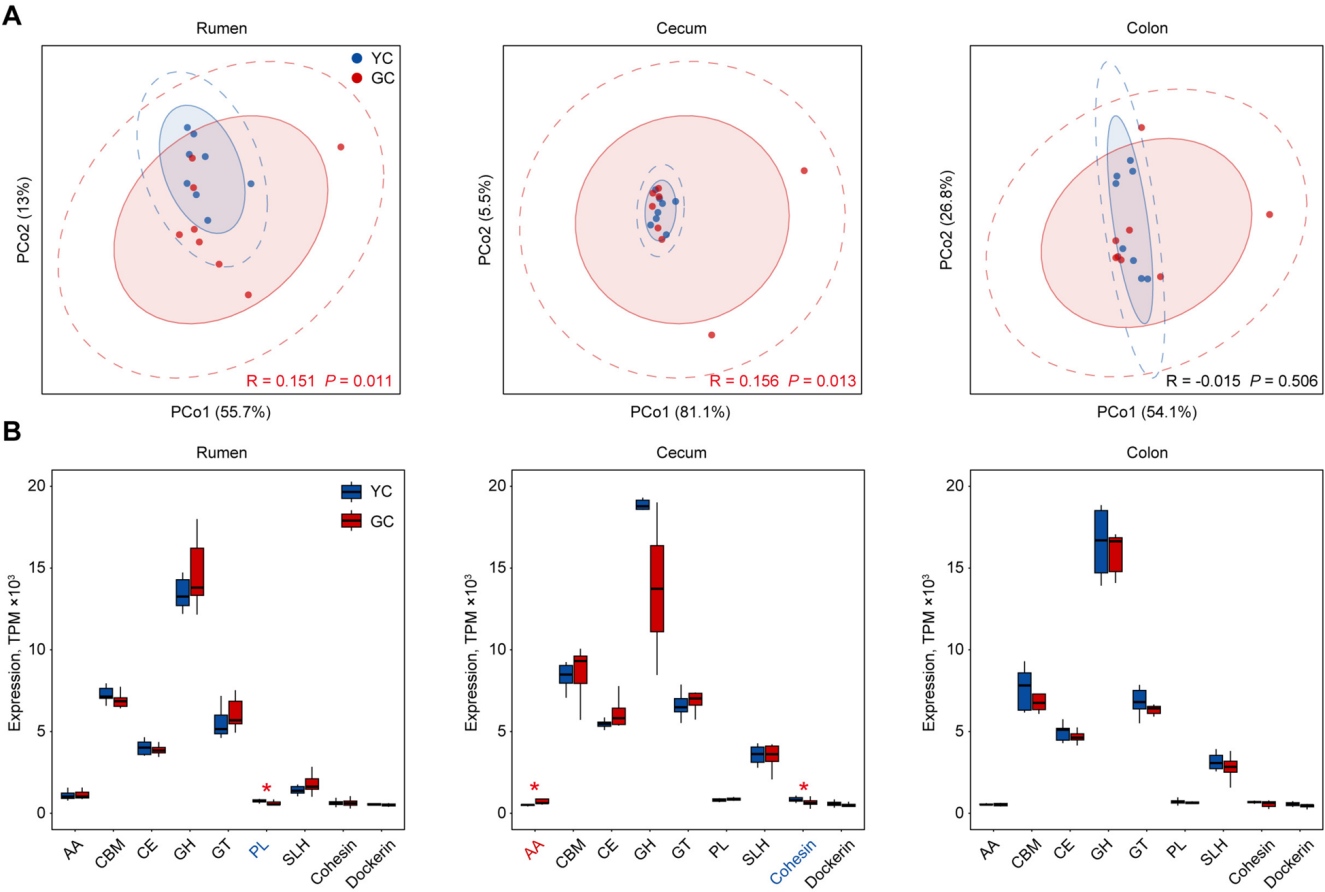
Expression of CAZymes between yellow cattle (YC) and gayal (GC). **A** PCoA plot of CAZyme gene expression profiles in the rumen, cecum, and colon microbiomes. **B** Bar plot showing the expression of total CAZyme families in the rumen, cecum, and colon. The center line of bar plot represents the median, the box indicates the interquartile range (IQR), and whiskers extend to 1.5 × IQR. Significance levels between groups are indicated as follows: ^*^P < 0.05

**Fig. 6:**
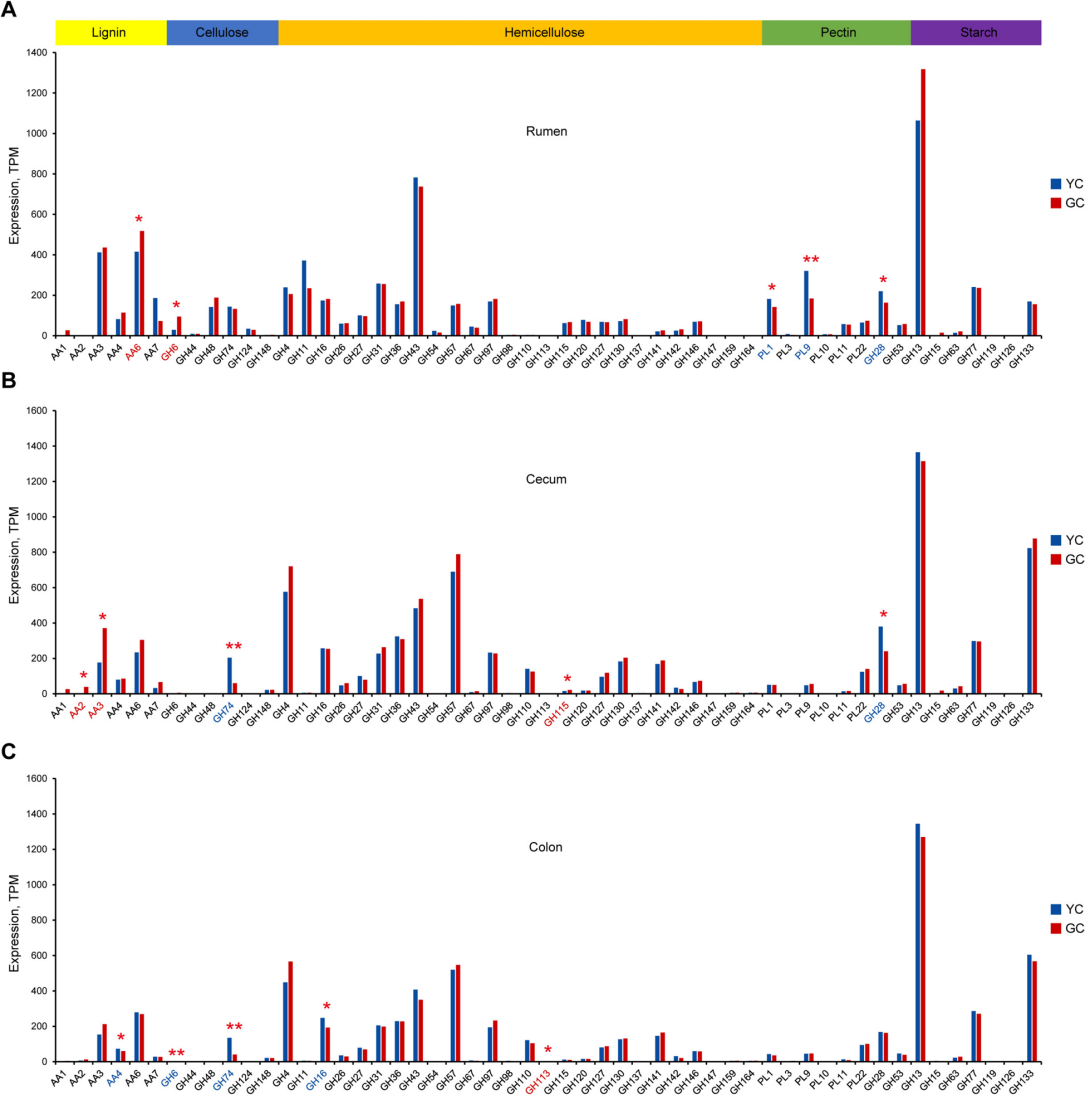
Comparative expression of key lignocellulose-degrading enzymes across gastrointestinal niches between yellow cattle (YC) and gayal (GC). **A**–**C** Expression of key CAZyme families are compared between YC and gayal GC in the rumen (**A**), cecum (**B**), and colon (**C**). Significance levels between groups are indicated as follows: ^*^P < 0.05, ^**^P < 0.01

The degradation of plant cell wall polysaccharides is primarily catalyzed by carbohydrate-active enzymes (CAZymes). In this study, a total of 3,572,080, 2,616,920, and 2,334,065 CAZyme genes were identified in the rumen, cecum, and colon, respectively. These three regions collectively contained nine enzyme types, including glycoside hydrolases (GH; 1,530,596, 1,206,433, and 1,071,439), carbohydrate-binding modules (CBM; 601,525, 421,180, and 380,043), glycosyltransferases (GT; 699,126, 383,532, and 343,126), carbohydrate esterases (CE; 404,249, 304,514, and 267,792), S-layer homology (SLH; 118,252, 155,841, and 140,844) domains, auxiliary activities (AA; 58,788, 24,924, and 21,533), polysaccharide lyases (PL; 70,336, 67,594, and 61,642), as well as cohesin (17,156, 21,684, and 19,408) and dockerin (64,375, 30,581, and 27,656) domains. Comparative analysis of CAZyme gene expression profiles revealed significant differences between yellow cattle and gayal in both the rumen (R = 0.151, *P* = 0.011) and cecum (R = 0.156, *P* = 0.013) microbiomes (Fig. 5A). In the rumen, yellow cattle exhibited significantly higher CAZyme expression of the total PL (*P* = 0.021) enzyme families (Fig. 5B). In the cecum microbiome, gayal showed a significant increase in the total AA expression (1.7-fold; *P* = 0.01), while yellow cattle had higher expression of the total cohesin modules (*P* = 0.05) (Fig. 5B). In contrast, no significant differences in the total CAZyme expression were detected between the two breeds in the colon (Fig. 5B). Notably, in the rumen, gayal exhibited specific enrichment of lignin-modification enzymes (AA6; 1.2-fold; *P* = 0.01) and cellulose-degrading enzymes (GH6; 3.3-fold; *P* = 0.015), while yellow cattle showed enrichment of pectin-degrading enzymes (PL1, PL9, and GH28) (Fig. 6A; Table S15). In the cecum, gayal was enriched in lignin-modification enzymes (AA2; 12-fold; *P* = 0.01 and AA3; 2.1-fold; *P* = 0.01) and hemicellulose-degrading enzymes (GH115; 1.5-fold; *P* = 0.038), while yellow cattle displayed higher expression of cellulose-degrading enzymes (GH74) and GH28 (Fig. 6B; Table S16). Within the colon, gayal was notably enriched in hemicellulose-degrading enzymes (GH113; 1.7-fold; *P* = 0.028), in contrast to yellow cattle, which showed increased expression of lignin-modification enzymes (AA4), GH6, GH74, and hemicellulose-degrading enzymes (GH16) (Fig. 6C; Table S17). These findings indicate that gayal possesses an enhanced capacity for carbohydrate degradation at the functional level, likely representing an evolutionary adaptation to a fibrous, lignocellulose-rich diet.

### Identification of core microorganisms encoding lignin-modification enzymes and their specialization in gayal

**Fig. 7:**
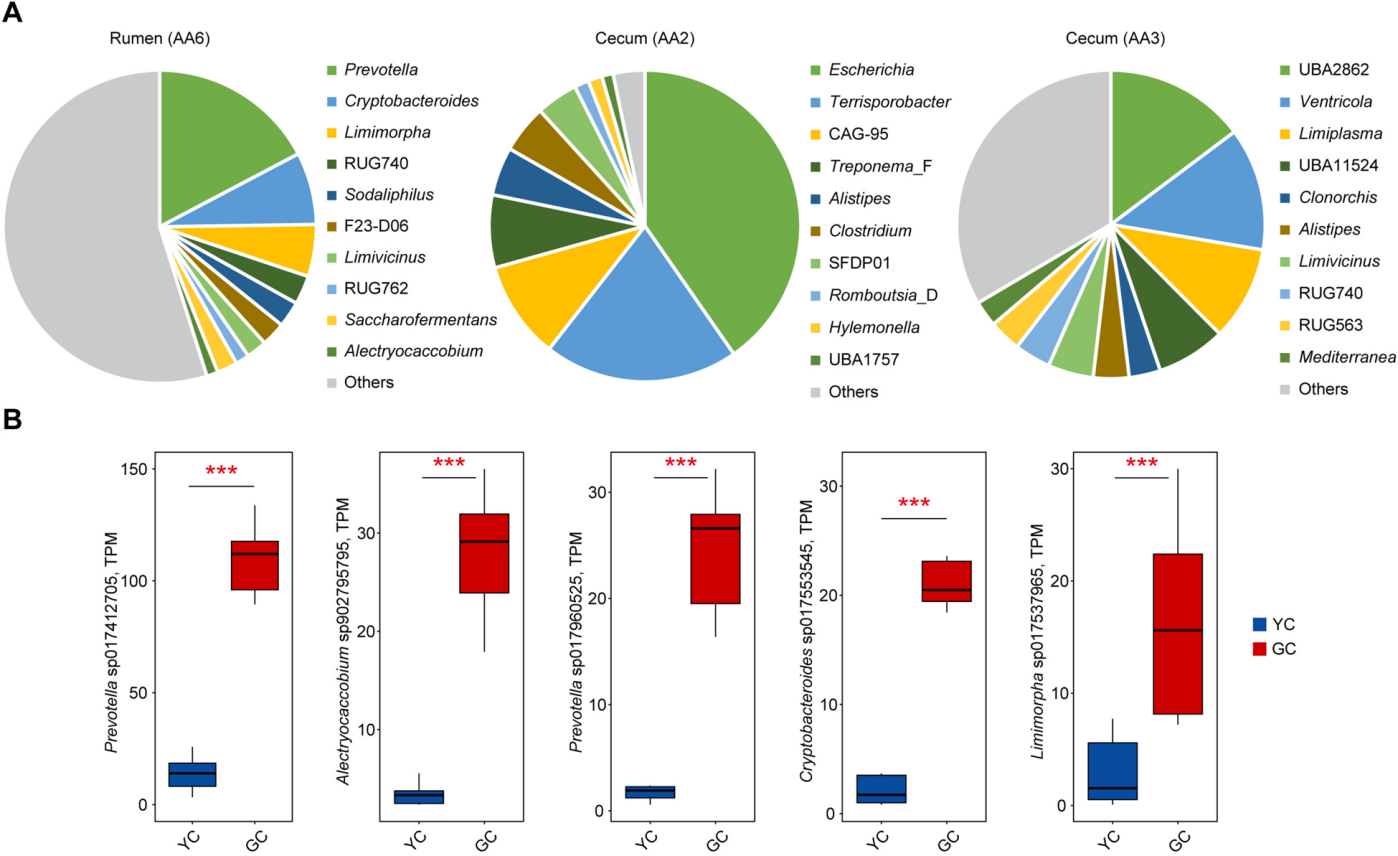
Taxonomic origins and differential expression of key lignin-modification enzymes (AA families) in the rumen and cecum. **A** Taxonomic composition of microbial genera encoding AA6 in the rumen, with AA2 and AA3 in the cecum, based on metatranscriptomic profiles. **B** Box plots illustrating the significant differences in gene expression of specific species encoding these rumen AA6 enzymes between yellow cattle (YC) and gayal (GC). Significance levels between groups are indicated as follows: ^*^P < 0.05, ^**^P < 0.01, and ^***^P < 0.001

As noted earlier, lignin-modification enzymes were specifically highly expressed in the rumen and cecum of gayal. To identify the key microbial taxa responsible for encoding these enzymes, particularly the AA6 family in the rumen and the AA2 and AA3 families in the cecum (Fig. 6), a homology-based analysis was conducted. The AA6 family enzymes (1,4-benzoquinone reductase) play a crucial role in removing lignin coatings that encapsulate polysaccharide networks [42]. A total of 41,929 microbial genes encoding AA6 were identified, distributed across 44 phyla, 931 genera, and 4,450 species. Among these AA6-encoding taxa in the rumen, *Prevotella* (17.3% of total expression) was the most dominant genus, followed by *Cryptobacteroides* (7.5%), *Limimorpha* (5.4%), and RUG740 (3%); genera with a relative abundance of less than 1.5% were grouped into the “Others” category (Fig. 7A). Notably, *Prevotella* sp017412705 and *Prevotella* sp017960525 were identified as major bacterial contributors enriched in the gayal rumen, exhibiting 6.7-fold (*P* < 0.001) and 14.7-fold (*P* < 0.001) higher expression compared to yellow cattle (Fig. 7B). Additionally, *Alectryocaccobium* sp902795795, *Cryptobacteroides* sp017553545, and *Limimorpha* sp017537965 showed significantly elevated expression (8.1-fold, 8.3-fold, and 5.6-fold, respectively; all *P* < 0.001) in the gayal rumen (Fig. 7B). Furthermore, a total of 87 and 4,028 microbial genes encoding AA2 and AA3, respectively, were identified in the cecum. These genes were distributed across 7 and 18 phyla, 25 and 175 genera, and 41 and 464 species for AA2 and AA3, respectively. AA2-encoding genes were primarily derived from *Escherichia* and *Terrisporobacter*, whereas AA3 was predominantly encoded by UBA2862, *Ventricola*, and *Limiplasma* (Fig. 7A). Among these, the expression of AA2-expressing species was notably low, with an average of only 7.5 TPM per sample, and the AA3-expressing species *Ventricola* sp017397695 showed a significant 1.2-fold upregulation (*P* = 0.038) in the gayal cecum. These findings indicate that the enhanced lignocellulose degradation in gayal is mediated by specialized rumen and cecum microbiomes through coordinated upregulation of key lignin-modification enzymes (AA6, AA2, and AA3), resulting in improved polysaccharide accessibility and metabolic efficiency.

## Discussion

In this study, we employed integrated metagenomic and metatranscriptomic analyses to elucidate the microbial mechanisms underlying the superior lignocellulose degradation capacity of gayal compared to yellow cattle fed an identical bamboo-based diet. Our multi-omics approach revealed distinct differences in microbial community composition and gene expression profiles that mechanistically explain the enhanced fiber utilization efficiency in gayal.

Consistent with previous reports [43–45], gayal demonstrated significantly superior growth performance and fiber digestibility compared to yellow cattle despite identical feed intake. This remarkable nutrition utilization efficiency is particularly noteworthy given the recalcitrant nature of bamboo, which consists of 47.9% cellulose, 17.6% hemicellulose (comprising 59.8% xylose, 17.9% glucose, 2.3% arabinose, and 0.43% galactose), and 27.1% lignin [46]. The superior performance of gayal on this recalcitrant substrate can be attributed to evolutionary adaptations in their gastrointestinal microbiome, shaped by long-term exposure to high-altitude, nutrient-poor, and fiber-rich forages environments [47]. Fermentation parameters further revealed that the distinct gastrointestinal microbiome in gayal drives a more efficient fermentative process, evidenced by significantly elevated concentrations of total VFAs and acetate.

High-altitude environments impose unique physiological challenges that have driven both host and microbial adaptations, resulting in enhanced metabolic efficiency and specialized enzymatic capabilities [47, 48]. The rumen serves as the primary fermentation chamber where the gayal’s superior fiber-degrading strategy is initiated. Compared to yellow cattle, the gayal rumen exhibited the most pronounced microbial and functional divergence, and a significant increase in bacterial and fungal alpha diversity. The enrichment of fiber-degrading taxa such as *Synergistota*, *Fibrobacterota*, and specific *Prevotella* species in gayal represents a co-evolutionary adaptation between host and microbiome that optimizes lignocellulose utilization. This specialized microbial architecture, driven primarily by highly active *Prevotella* species that strongly upregulate AA6 and GH6, directly facilitates the observed metabolic outcome by enhancing lignocellulose accessibility. The prominence of AA6 enzymes (1,4-benzoquinone reductases) represents a particularly important finding, as they facilitate the initial disruption of lignin-carbohydrate complexes through quinone-mediated reactions [43]. This coordinated action is supported by the significantly higher cellulase and xylanase activities, which collectively drive the elevated production of acetate and butyrate, setting the stage for efficient downstream fermentation.

The enhanced fermentative performance extends beyond the rumen into the hindgut, a trait critical for maximizing energy harvest from a high-fiber diet. This was evidenced by the continued elevation of total VFAs in gayal, particularly acetate in both cecum and colon, and propionate and butyrate in the colon. The distinct enrichment of Fibrobacterota in both the cecum and colon of gayal indicates a sustained, robust cellulolytic capacity, which is supported by the significantly higher cellulase and xylanase activities throughout the hindgut. Specifically, the upregulation of mannanase in the cecum and arabinofuranosidase in the colon directly targets the hemicellulose composition of the bamboo diet. Furthermore, the heightened expression of AA2 and AA3 in the cecum suggests a continuation of lignin modification processes to maximize the breakdown of recalcitrant fibers. Ultimately, the convergence of these compartment-specific microbial and enzymatic adaptations ensures that gayal captures additional energy that escaped rumen digestion.

To elucidate the microbial strategy for degrading recalcitrant fibers, we investigated the underlying enzymatic mechanisms and found that AA6 enzymes were broadly distributed across diverse taxa, indicating a community-wide strategy for lignin deconstruction. The elevated expression of AA6, AA2, and AA3 in gayal provides direct mechanistic insights into how lignin is modified under anaerobic conditions. Rather than complete depolymerization, these enzymes modify the lignin polymer and cleave its linkages to polysaccharides, thereby enhancing substrate accessibility for hydrolases. Specifically, AA6 enzymes generate reactive quinone intermediates that drive Fenton-like reactions, producing hydroxyl radicals which cleave lignin-polysaccharide bonds and expose cellulose fibers for enzymatic attack [49, 50]. This system, reliant on microbially derived Fenton reagents, significantly improves lignocellulose accessibility for a broader suite of cellulases and hemicellulases.

This capacity for anaerobic lignin modification, while distinct from aerobic degradation, shares functional parallels with mechanisms in other efficient systems like termite guts [48]. The coordinated action of multiple auxiliary activity families represents a sophisticated enzymatic cascade that enhances substrate accessibility. In our study, the genus *Prevotella* emerged as a central player, consistent with its established role as a versatile polysaccharide degrader in ruminants [51]. The specific enrichment of *Prevotella* species encoding high levels of AA6 in gayal suggests their evolutionary specialization for enhanced lignin modification. Furthermore, the presence of *Cryptobacteroides, Limimorph*a, and other fiber-degrading genera indicates a consortium-based approach, where different taxa contribute complementary enzymatic functions to achieve efficient lignocellulose deconstruction [52, 53].

While our integrated approach provides potential mechanistic insights, several limitations warrant consideration. Whilst our profiling detected the presence of anaerobic fungi and protozoa, which are known to be potent lignocellulose degraders, their specific contributions to the process remain unresolved due to their low relative abundance and limitations in our functional annotation. Although we measured enzyme activities, transcript levels may not fully equate to functional protein abundance due to post-translational regulation. Our sample size, while sufficient to reveal significant differences, highlights the need for validation in independent cohorts. Future studies integrating ITS/18S sequencing, proteomics, and cultivation-based methods are essential to fully delineate the roles of all microbial players and validate the proposed metabolic model.

Collectively, our findings position the gayal microbiome as a valuable bioresource for novel biotechnological applications in lignocellulose processing. The identification of highly active lignin-modifying enzymes and their associated microbial hosts provides targets for enzyme mining and microbial consortium development. These findings have implications for improving fiber utilization in livestock production through microbiome-based interventions such as probiotic supplementation, selective breeding for beneficial microbial traits, or the development of enzyme additives inspired by the AA-mediated pathways identified in gayal.

## Conclusions

This comprehensive metagenomic and metatranscriptomic investigation reveals the molecular basis for gayal’s superior lignocellulose degradation capacity compared to yellow cattle. The key findings demonstrate that gayal harbors specialized microbial communities enriched in fiber-degrading taxa and exhibits significantly upregulated expression of lignin-modifying auxiliary activity enzymes, particularly AA6, AA2, and AA3 families. The AA6-centered enzymatic system facilitates the disruption of lignin-carbohydrate complexes through quinone-mediated reactions, thereby enhancing polysaccharide accessibility and supporting superior growth performance on fibrous diets. These mechanistic insights advance our understanding of ruminant adaptation to challenging dietary environments and highlight the gayal microbiome as a valuable genetic and microbial resource. The findings provide a foundation for developing microbiome-based strategies to enhance fiber utilization in livestock production, including the screening of gayal-derived microbial consortia, design of targeted AA-enzyme additives, optimization of feed pretreatment processes inspired by the gayal’s digestive strategy, and implementation of precision microbiome interventions aimed at enriching for these specialized lignocellolytic taxa. Future research should focus on translating these discoveries into practical applications for sustainable livestock production and industrial lignocellulose bioprocessing.

## Supplementary Information

Additional file 1: Table S1. Ingredients and chemical composition of the diet (%, DM basis). Table S2. Growth performance and apparent digestibility of yellow cattle (YC) and gayal (GC). Table S3. Comparison of rumen, cecum, and colon fermentation parameters between yellow cattle (YC) and gayal (GC). Table S4. Analysis of cellulase and hemicellulase activities in the rumen, cecum, and colon of yellow cattle (YC) and gayal (GC).

Additional file 2: Table S5. The assembly results for the total of 96 metagenomic and metatranscriptomic samples from the rumen, cecum, and colon of yellow cattle (YC) and gayal (GC). Table S6. Relative abundance of top 30 abundant microbial phyla across in the rumen of yellow cattle (YC) and gayal (GC). Table S7. Relative abundance of top 30 abundant microbial phyla across in the cecum of yellow cattle (YC) and gayal (GC). Table S8. Relative abundance of top 30 abundant microbial phyla across in the colon of yellow cattle (YC) and gayal (GC). Table S9. Relative abundance of top 35 abundant microbial genera across in the rumen of yellow cattle (YC) and gayal (GC). Table S10. Relative abundance of top 35 abundant microbial genera across in the cecum of yellow cattle (YC) and gayal (GC). Table S11. Relative abundance of top 35 abundant microbial genera across in the colon of yellow cattle (YC) and gayal (GC).

Additional file 3: Table S12. Top 20 KEGG pathway enrichment in the rumen of yellow cattle (YC) and gayal (GC) based on metatranscriptomic profiling. Table S13. Top 20 KEGG pathway enrichment in the cecum of yellow cattle (YC) and gayal (GC) based on metatranscriptomic profiling. Table S14. Top 20 KEGG pathway enrichment in the colon of yellow cattle (YC) and gayal (GC) based on metatranscriptomic profiling.

Additional file 4: Table S15. Expression of key lignocellulose-degrading enzymes between yellow cattle (YC) and gayal (GC) in the rumen. Table S16. Expression of key lignocellulose-degrading enzymes between yellow cattle (YC) and gayal (GC) in the cecum. Table S17. Expression of key lignocellulose-degrading enzymes between yellow cattle (YC) and gayal (GC) in the colon.

Additional file 5: Fig. S1. Differential beta diversity of rumen microbial communities between yellow cattle (YC) and gayal (GC) across multiple kingdoms.

Additional file 6: Fig. S2. Beta diversity of cecal and colonic microbial communities between yellow cattle (YC) and gayal (GC) across multiple kingdoms.

Additional file 7: Fig. S3. Taxonomic composition of the rumen and hindgut microbiota in yellow cattle (YC) and gayal (GC) across multiple kingdoms.

## Data Availability

Raw sequence reads for all metagenomic and metatranscriptomic samples from this study are available under European Nucleotide Archive (ENA) project PRJNA1301107. Other datasets generated and analyzed during the current study are available from the corresponding author on reasonable request.

## Abbreviations

AA: Auxiliary activities
ADF: Acid detergent fiber
ADG: Average daily gain
ADL: Acid detergent lignin
CP: Crude protein
CAZymes: Carbohydrate-active enzymes
DMI: Dry matter intake
GC: Gayal
GH: Glycoside hydrolases
KEGG: Kyoto Encyclopedia of Genes and Genomes
OM: Organic matter
NDF: Neutral detergent fiber
PCoA: Principal coordinates analysis
TPM: Transcripts per million
VFA: Volatile fatty acid
YC: Yellow cattle
