# Supplementary figures and images for: Comparative metagenomic and metatranscriptomic analyses reveal the role of the gayal rumen and hindgut microbiome in high-efficiency lignocellulose degradation
Source: J Anim Sci Biotechnol. 2026 Feb 2;17:18. doi: 10.1186/s40104-025-01335-1 (PMC12862909; doi:10.1186/s40104-025-01335-1)

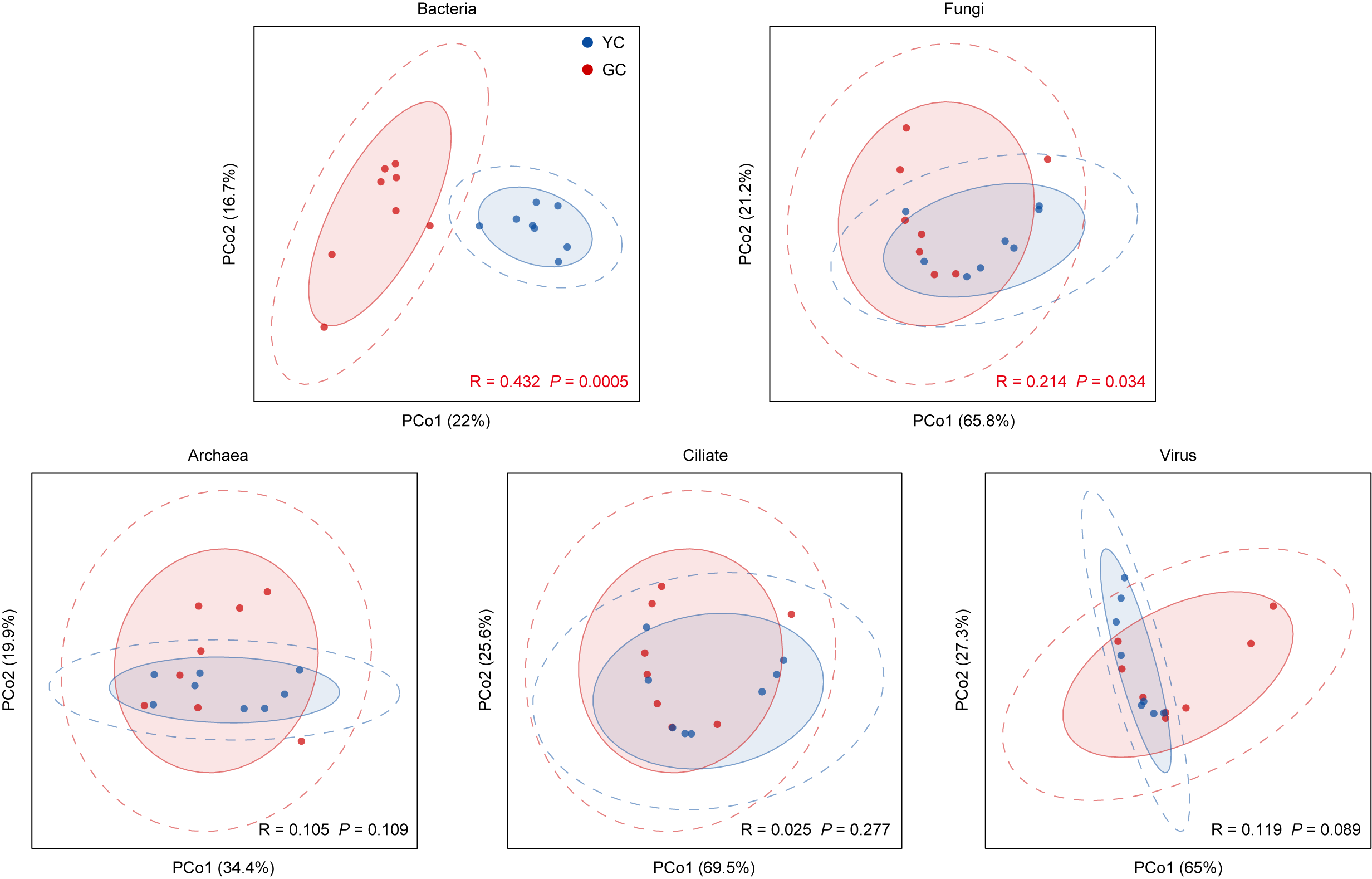

Supplement: Supplementary file 5 — Additional file 5: Fig. S1. Differential beta diversity of rumen microbial communities between yellow cattle (YC) and gayal (GC) across multiple kingdoms. [file 40104_2025_1335_MOESM5_ESM.tif]

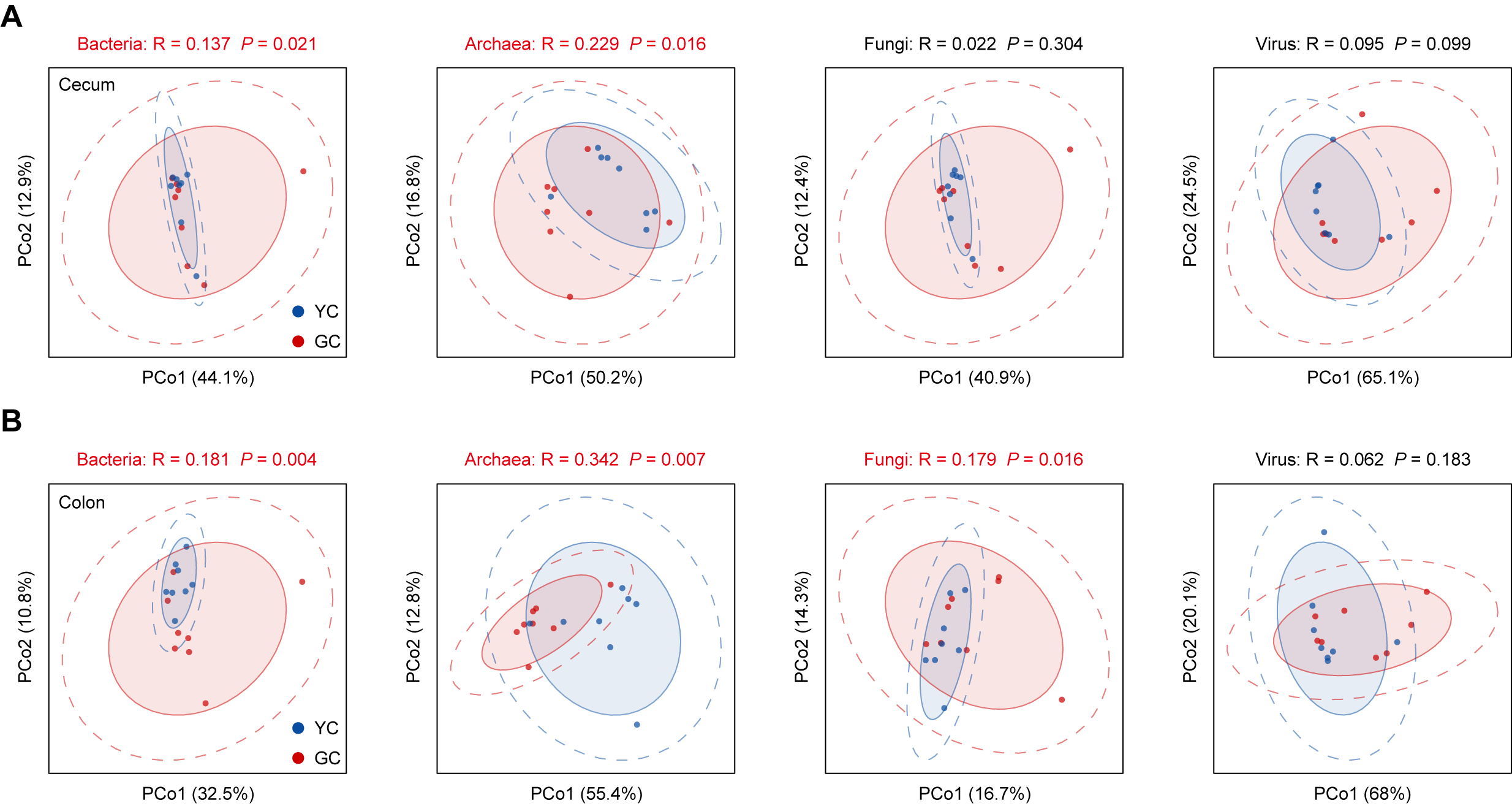

Supplement: Supplementary file 6 — Additional file 6: Fig. S2. Beta diversity of cecal and colonic microbial communities between yellow cattle (YC) and gayal (GC) across multiple kingdoms. [file 40104_2025_1335_MOESM6_ESM.tif]

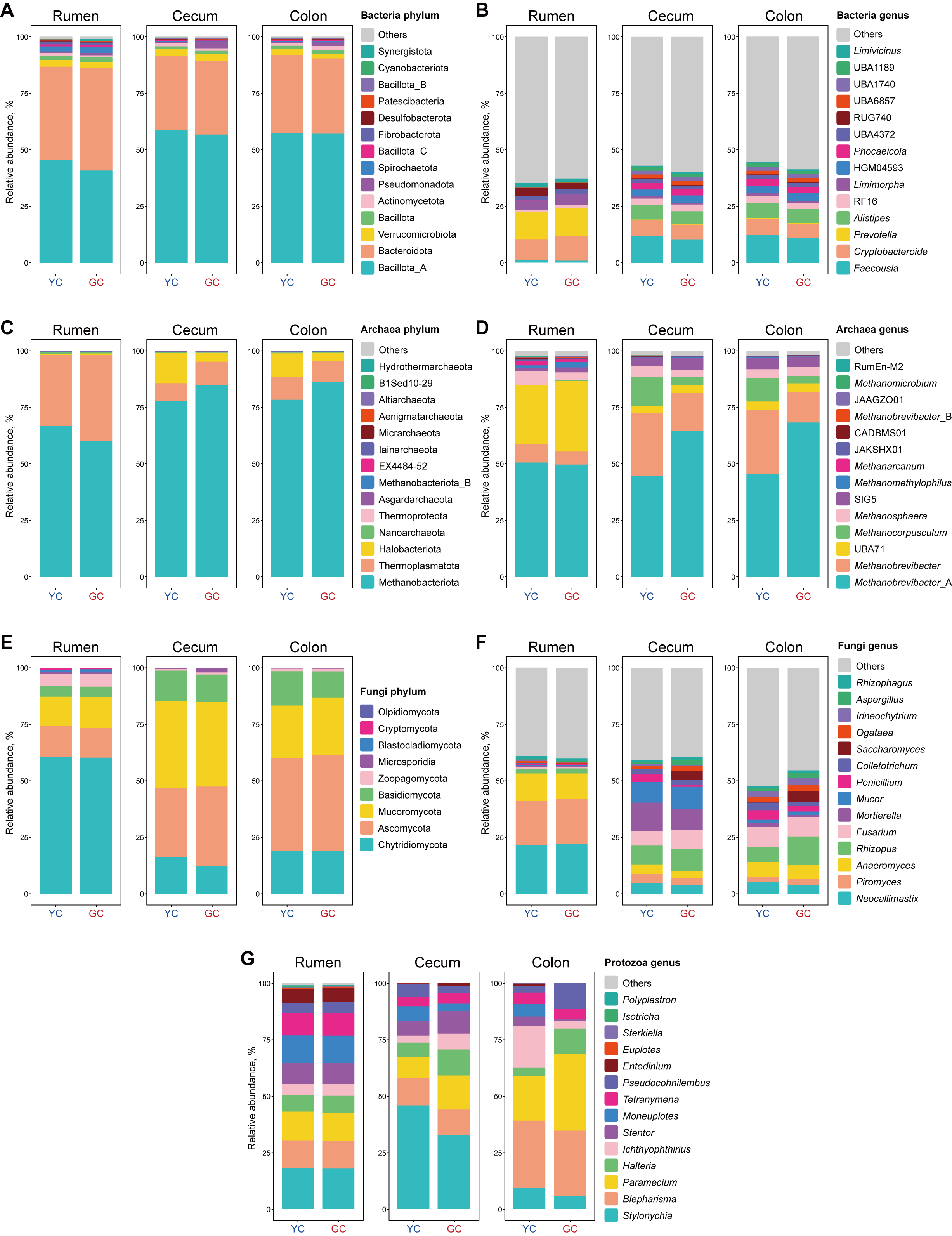

Supplement: Supplementary file 7 — Additional file 7: Fig. S3. Taxonomic composition of the rumen and hindgut microbiota in yellow cattle (YC) and gayal (GC) across multiple kingdoms. [file 40104_2025_1335_MOESM7_ESM.tif]
